# Autochthonous Ascariasis, Mississippi, USA

**DOI:** 10.3201/eid3004.240176

**Published:** 2024-04

**Authors:** Charlotte V. Hobbs, James Matthew Rhinewalt, Irene Arguello, Lacy Malloch, Lora Martin, William M. Poston, Paul Byers, Richard S. Bradbury

**Affiliations:** University of Alabama at Birmingham/Childrens of Alabama, Birmingham, Alabama, USA (C.V. Hobbs);; Children’s of Mississippi/University of Mississippi Medical Center, Jackson, Mississippi, USA (C.V. Hobbs, I. Arguello, L. Malloch, L. Martin);; Internal Medicine and Pediatric Clinic, New Albany, Mississippi, USA (J.M. Rhinewalt);; Baptist Hospital Systems, New Albany (W.M. Poston);; Mississippi State Department of Health, Jackson (P. Byers);; James Cook University, Townsville, Queensland, Australia (R.S. Bradbury)

**Keywords:** Ascariasis, *Ascaris lumbricoides*, Mississippi, United States, zoonoses, swine, parasites, helminths

## Abstract

We describe a case of a 2-year-old child who expelled a single adult female *Ascaris lumbricoides* worm. The patient is from a rural county in Mississippi, USA, with no reported travel outside of the United States. The caregivers in the home practice good sanitation. Exposure to domestic pigs is the likely source of infection.

A reported increase of hookworm and strongyloidiasis transmission in rural Alabama, USA, in 2017 (*1*) has led to more interest in isolated cases of autochthonous transmission of soil-transmitted helminths in the southeastern United States. This increased transmission and interest led to several small- and large-scale surveys of soil-transmitted helminths and other parasitic diseases in Mississippi (*2*–*4*), Alabama (*5*), and Texas (*6*). No cases of ascariasis were identified in those surveys. However, highly endemic porcine ascariasis is present in some farmed pigs in the United States (*7*). Sporadic reports have been documented of autochthonous ascariasis cases and case clusters in northeastern states (*8*), and *Ascaris lumbricoides* roundworm–mediated Löffler syndrome (eosinophilic pneumonitis) has been reported in Louisiana over the past decade (*9*). Those autochthonous ascariasis cases represented spillover infections to humans from pigs. We describe a case of zoonotic ascariasis from New Albany in Union County, Mississippi.

A previously healthy 2-year-old girl was brought to her local pediatrician with complaints of abdominal cramping for 2 weeks, loose stools (without blood or mucus), and a decreased appetite. The family was originally from Mexico but had lived in the United States for 13 years. Neither the patient nor her twin sister had been outside of the United States. The family lived on a farm with pigs, and both children reportedly ate dirt from the house plants. The mother found a motile worm in the patient’s diaper, filmed the worm, and then discarded the diaper and worm. 

We identified the helminth from the video (Video) as an adult female *A. lumbricoides* worm because of the characteristic size, shape, reddish-orange color, and a pointed rather than recurved tail. The patient was treated by her pediatrician with ivermectin (1 dose of a 3 mg tablet) because albendazole was not available and mebendazole was not covered by the patient’s insurance. We performed automated complete blood counts by using an in-office hematology analyzer (without eosinophil count capacity). The patient was not anemic (hemoglobin 11.8 g/dL [reference range for age 11–13.7 g/dL]; mean corpuscular volume 80.4 fL [reference range for age 75–86 fL]). We treated the family members as a precautionary measure. We obtained stool samples from the patient within 24 hours of treatment but detected no eggs on Kato–Katz microscopic smear. The patient did not expel any additional worms. We followed the patient clinically with complete blood counts, and her symptoms resolved without complication.

**Video:** Adult, motile, *Ascaris lumbricoides* worm in the diaper of a 2-year-old patient expelled through the rectum. The worm was found by the patient’s mother, who videoed the worm before disposing of the diaper and worm.

The Mississippi State Department of Health conducted field visits to the patient’s home, but the family’s pigs had been sent to slaughter. The family has 2 flush toilets in their home used for all defecation and disposal of feces. The Mississippi State Department of Health counselled all family members on handwashing, especially after contact with soil where pigs had defecated.

This case represents an autochthonous acquisition of ascariasis in the southeast United States. Only 1 worm was expelled by the patient, even after treatment, and the absence of eggs when treatment occurred suggests that this patient harbored a single adult worm infection. The lifespan of adult *A. lumbricoides* worms within human hosts is up to 2 years, and eggs may remain viable in soil for up to 10 years (*10*). The patient’s family had been living in the United States for 13 years, and no promiscuous defecation was occurring in the child’s environment. However, the child lived near domestic pigs, which is a common zoonotic origin for this infection.

Ascariasis is often asymptomatic or subclinical, although abdominal pain, distension, and wasting may occur (*7*–*10*). Adult worms migrating in the intestinal tract may obstruct the bile or pancreatic ducts, leading to cholecystitis or pancreatitis (*10*). Occasionally, migrating adult worms may be expelled through the rectum or emerge from the nose or mouth. In patients with heavy worm infections, bowel obstruction, intussusception, volvulus, and small bowel perforation may occur (*10*). Heavy infections may also cause malabsorption and stunting with consequent vitamin deficiencies, growth retardation, altered immunity, and impaired cognition (*7*,*10*). A larva migrans syndrome may be observed, caused by immune-mediated responses to the visceral migration of *A. lumbricoides* worm larvae through the lungs and appearing as Löffler syndrome (*9*).

Confusion exists over the taxonomic status of *Ascaris* helminths in pigs and humans. The genus *Ascaris* was once split into 2 species, *A. suum* and *A. lumbricoides*, but modern genotyping methods have determined that the 2 categories are instead separate genotypes of the same species, *A. lumbricoides* (*7*). Genotypic surveillance of adult *A. lumbricoides* worms from farmed pigs in Iowa found 10 haplotypes present, including those belonging to *A.* *lumbricoides* (human), *A.* *suum* (pig), and hybrid genotypes (*7*).

In summary, we describe a case of likely zoonotic autochthonous human ascariasis acquired in rural northern Mississippi. Sporadic ascariasis cases in the United States are most often zoonotic in origin, with exposure to pigs, or soil contaminated with pig feces, as the primary risk factor.
