# Supplementary figures and images for: Autochthonous Ascariasis, Mississippi, USA
Source: Emerg Infect Dis. 2024 Apr;30(4):821–3. doi: 10.3201/eid3004.240176 (PMC10977833; doi:10.3201/eid3004.240176)

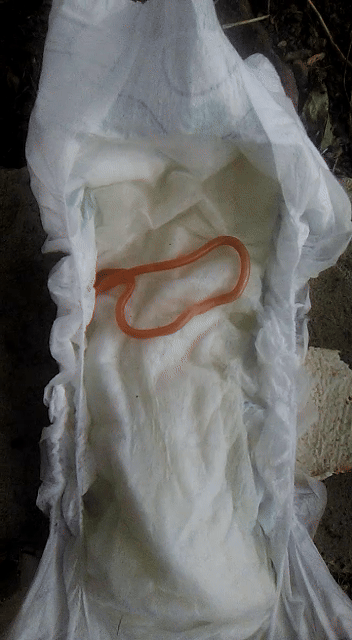

Supplement: Supplementary file 1 [file 24-0176-V.gif]
